# Alterations in Dietary Behavior, Appetite Regulation, and Health-Related Quality of Life in Youth with Obesity in Germany during the COVID-19 Pandemic

**DOI:** 10.3390/nu15132936

**Published:** 2023-06-28

**Authors:** Nora Struckmeyer, Torben Biester, Olga Kordonouri, Chantal Weiner, Evelin Sadeghian, Cathrin Guntermann, Kerstin Kapitzke, Jantje Weiskorn, Laura Galuschka, Kisa von Stuelpnagel, Daniela Meister, Karin Lange, Thomas Danne, Felix Reschke

**Affiliations:** 1 Center for Pediatric Diabetology, Endocrinology, and Metabolism, Children’s Hospital AUF DER BULT, 30173 Hannover, Germany; 2 Department for Sports Science, University of Hildesheim, 31141 Hildesheim, Germany; 3 Medical Psychology, Hannover Medical School, 30625 Hannover, Germany

**Keywords:** nutritional content, hunger control, children with overweight, life quality, eating habits, SARS-CoV-2

## Abstract

Background: This study aimed to evaluate the impact of the COVID-19 pandemic on the nutritional patterns, eating behavior, dietary content, and health-related quality of life (HrQoL) of adolescents with preexisting obesity. Methods: Anthropometric and metabolic parameters were measured, and validated questionnaires on eating habits, nutritional content, and HrQoL were administered to 264 adolescents with obesity during the COVID-19 pandemic (June 2020–June 2022) and 265 adolescents with obesity before the pandemic (from June 2017 to June 2019). Results: Both study cohorts were comparable in age and sex distribution. Significant differences were found between the COVID-19 and pre-COVID-19 cohorts in HOMA-index (3.8 (interquartile range [IQR])): 3.3; 4.1) vs. 3.2 (IQR: 2.8; 3.5, *p* < 0.001), total cholesterol (208.8 mg/dL (IQR: 189.9; 214.5) vs. 198.5 mg/dL (IQR: 189.5; 207.4), *p* < 0.001), and GPT (93.4 (IQR 88.7; 96.5) vs. 72.8 U/L (IQR 68.9; 75.7), *p* < 0.001). The COVID-19 cohort reported significantly higher consumption of obesity-promoting food components, such as soft drinks, meat, sausages, fast food and delivery food, chocolate, and sweets. There was also a significant decrease in cognitive hunger control (*p* = 0.002) and an increase in distractibility potential (*p* = 0.001) while eating. HrQoL was significantly lower in the COVID-19 cohort (*p* = 0.001). Conclusions: This study reveals the adverse associations of exposure to the public health measures during the COVID-19 pandemic with nutrition, dietary content, and HrQoL in adolescents with preexisting obesity. These findings underscore the importance of tailored preventive and treatment strategies for addressing the specific challenges of disruptive events such as pandemics, especially in population-based context.

## 1. Introduction

Adolescence is a critical period characterized by rapid growth, physical development, and significant changes in dietary patterns. Proper nutrition during this period is essential to support optimal growth, development, and overall health. However, adolescents often face unique challenges in achieving a healthy diet due to various factors, such as increased autonomy in food choices, exposure to unhealthy food environments, and peer influences.

Furthermore, dietary habits established during adolescence tend to track into adulthood, influencing the risk of developing chronic diseases later in life. The establishment of unhealthy dietary patterns during this period can have long-lasting effects on overall health outcomes. Poor nutrition and unhealthy eating habits during adolescence can contribute to an increased risk of weight gain and related health issues [1,2,3]. Childhood obesity has become a prevalent issue in numerous countries worldwide, defined as having a body mass index (BMI) above the 95th percentile for a child’s age and sex. Factors contributing to its development include genetics, a sedentary lifestyle, and unhealthy eating habits. In this regard, the significance of food components, portion sizes, and eating behavior is crucial [4,5,6,7]. Eating behavior and cognitive appetite control play a critical role in the development of childhood obesity. Poor cognitive appetite control can result in overeating and weight gain. Various interfering and distracting factors, such as atmosphere, electronic devices, and TV, can also affect food intake and increase the risk of childhood obesity by causing mindless eating and overeating [8,9]. In addition, the extent of unpleasant hunger-associated feelings experienced during fasting periods, particularly among young people, can also influence the development of obesity [10,11]. This can result in overeating later, leading to a calorie surplus and eventual weight gain.

To prevent childhood obesity, it is necessary to consider several factors. A comprehensive approach that takes into account the importance of food components, food quantities, eating behavior, cognitive appetite control, interfering and distracting factors like atmosphere, etc., is required. Encouraging healthy eating habits, regular physical activity, and healthy lifestyle choices from an early age is essential to help children maintain a healthy weight and prevent obesity-related health issues [12,13]. Childhood obesity is a significant public health challenge that can lead to a range of negative health outcomes [14]. In addition to the increased risk of developing secondary diseases such as diabetes, insulin resistance, and lipid metabolism disorders, obesity in youth can also lead to a higher risk of developing other chronic health conditions later in life, such as cardiovascular disease and certain cancers [15] Health-related quality of life (HrQoL) is an important consideration in the management of pediatric obesity. HRQoL refers to the impact of a health condition on a person’s physical, emotional, and social functioning and well-being [16,17]. Childhood obesity can have negative effects on a child’s HRQoL due to a range of factors, including social stigmatization, physical limitations, and reduced self-esteem [18]. Accompanying risk factors for the development and persistence of childhood overweight are an altered day/night rhythm with shortened or altered sleep duration and increased screen time [19]. 

During the COVID-19 pandemic, measures were implemented to prevent the spread of the SARS-CoV-2 virus, including restrictions on contact and movement. In Germany, the first wave of COVID-19 led to the closure of schools, public sports fields, and gyms from mid-March 2020 to early June 2020, and club sports were prohibited. Adolescents were not provided with support in their daily routines, dietary management, and physical activity during this period. The second lockdown occurred from November 2020 to May 2021 and, again, all people were asked to stay at home and sports facilities were closed. Club sports could not take place, and school cafeterias were also closed. There was no official support for nutrition and daily life during this time. In the third lockdown, from November 2021 to March 2022, club sports were prohibited, and although schools were open, many young people did not have access to school lunches or other nutritional services in many parts of Germany [20]. 

The COVID-19 pandemic caused a significant shift in the daily routines of children and young people, resulting in changes in sleep patterns, increased screen time, and reduced access to structured meals like school lunches. As a consequence, many young people, including those with pre-existing obesity, experienced weight gain and a decline in their HrQoL [21]. However, the extent to which these lockdown measures impacted this group of children is unknown. Therefore, this cross-sectional study aims to examine the associations between the COVID-19 pandemic and nutritional content, and HrQoL of children and adolescents referred from their primary care physician for the treatment of obesity to a certified tertiary care treatment center for pediatric obesity.

The COVID-19 pandemic and its associated restrictions have likely resulted in significant changes to the eating patterns, nutritional content, eating behavior, and health-related quality of life of adolescents with pre-existing obesity. The aim of this study is to evaluate possible changes, including altered dietary habits, the increased consumption of unhealthy foods, and negative impacts on overall well-being in youth with pre-existing obesity when compared to the pre-pandemic period. 

## 2. Materials and Methods

### 2.1. Data Collection

The Children’s Hospital AUF DER BULT in Hanover maintains one of the largest centers for the care of childhood obesity in northern Germany. The study model compares two cohorts of children and adolescents aged 8.0–17.9 years with obesity, one participating in the treatment program before the COVID-19 pandemic (assigned as “pre-COVID-19”—from 1 June 2017 to 30 June 2019) and one joining during the pandemic (assigned as “COVID-19”—from 1 June 2020, to 30 June 2022). The children and adolescents were identified based on having a BMI z-score greater than 2.0 and being sent to the outpatient obesity department “AUF DER BULT” by their GP or pediatrician for treatment. To ensure the comparability of the two cohorts, there were no differences in the referral, selection, examination and data-collection procedures between the two time periods. All children underwent a physical examination and received a standardized laboratory analysis. The clinical examination was conducted by a pediatrician specialized in pediatric obesity and included both a clinical physical examination and standardized laboratory analysis. The laboratory analysis involved various parameters, including leukocytes, erythrocytes, hemoglobin, hematocrit, thrombocytes, HbA1C value, fasting glucose, fasting insulin, HOMA (GOT), GPT, creatinine, total cholesterol, LDL cholesterol, HDL cholesterol, urea, triglycerides, gamma GT, and TSH. For the blood count and HbA1C value, EDTA collection tubes were used, and analysis was performed using flow cytometry. Fasting glucose was collected with fluoride EDTA tubes and measured through spectrometry. The remaining parameters were collected using lithium heparin gel tubes. Insulin and HOMA levels were determined using chemiluminescence immunoassay (CLIA). All collection tubes used were S-monovettes^®^ from Sarstedt (Germany). In addition, all study participants recorded their dietary behavior and content, as well as their HrQoL, using standardized questionnaires. 

### 2.2. Nutritional Behavior

Dietary content was measured using the standardized Kinder—Food Frequency List (K-FFL) [22]. K-FFL is a quantitative frequency questionnaire and captures a total of 37 items on the frequency of consumption of eight different food groups (cereal products/potatoes; vegetables/salad; fruits; dairy products; meat/fish products; fats; beverages; and sweets/fast food) in food-specific portion sizes. Preset response categories included “3–5 servings per day”, “1–2 servings per day”, “4–6 servings per week”, “1–3 servings per week”, and “rarely or never”. For ease of the presentation of results, dietary patterns were grouped into healthy and unhealthy consumption frequencies. The response categories “3–5 servings per day”, “1–2 servings per day”, and “4–6 servings per week” were combined into the “healthy frequency of consumption” category for healthy foods and the “unhealthy frequency of consumption” category for unhealthy foods. Correspondingly, the response categories “1–3 servings per week” and “rarely or never” were combined into the category “unhealthy frequency of consumption” for healthy foods and “healthy frequency of consumption” for unhealthy foods according to a previously reported evaluation model [23]. The evaluation was limited to pre-selected obesity-relevant foods (beverages, meat products, cheese or dairy products, fish, rice/pasta, various types of bread, fruit, vegetables, fast food, and confectionery).

The K-FEV questionnaire is a validated questionnaire used to evaluate the eating behavior of adolescents. It was modified and translated into German using the “Three-Factor-Eating-Questionnaire” [24]. The questionnaire consists of a total of 51 items (Supplementary Table S1). The surveyed questions register three scales of the FEV on the scales of cognitive control of eating behavior (21 items), disruptability of eating behavior (16 items) and experienced feelings of hunger (14 items). The contents of the scales are shown in Supplementary Table S1. High values on the scale “cognitive control” characterize subjects with strongly restrained eating behavior. Low values indicate a spontaneous, unrestrained eating behavior regulated by the internal signals of autonomous appetite and satiety regulation. High values in the scale “uncontrolled eating behavior” are achieved by subjects with an increased disturbability in their eating manner. Low distractibility is characterized by low scores. Higher scores are associated with greater food intake and, accordingly, with higher body weight. Successful weight reduction is made more difficult by increased interference. High values on the scale “hunger sensations while fasting” characterize strongly experienced feelings of hunger, often perceived as disturbing, which motivate increased food intake. The questions contain a total of 51 items in the form of statements in the first person, most of which can be answered with “agree” or “disagree”. For the remaining questions, patients are offered four different answer options (“always”, “often”, “rarely”, “never”), and can choose which best applies to them. The items were coded according to the evaluation key and added up to three scale sum values (maximum 58) [25,26]. K-FFL and K-FEV are the recommended assessment tools of the German Obesity society (www.adipositas-gesellschaft.de, accessed on 27 June 2023). To examine the differences within the entire group, the data were analyzed collectively. Additionally, to investigate the potential influence of sex as a factor, the data were stratified by sex and analyzed separately for males and females. To allow for a more comprehensive evaluation of the observed data and their relevance in relation to established norms, we additionally compared our findings with established reference data [27]. This will provide insights into how children with obesity may differ in their eating habits compared to those within the normal weight range.

### 2.3. Health-Related Quality of Life (HrQoL)

The KINDL-R is a validated questionnaire for assessing health-related quality of life in children and adolescents that can be used in clinical and normal populations [28]. It consists of a Likert-Scale of 24 items, with a total of 6 dimensions of quality of life: physical well-being, psychological well-being, self-esteem, well-being in the family and in relation to friends, and well-being at school. KINDL-R meets the requirement of taking into account the individual child’s developmental stage, with different versions for different age groups. Respondents can answer the items using a five-level response category (never, rarely, sometimes, often, always). From the responses, an overarching measure of health-related quality of life can be calculated from all 24 items [29]. Since school did not occur consistently during the observation period, the HrQoL in school subscale of this questionnaire was not included in this evaluation. Additionally, the data for health-related quality of life (HrQoL) will be compared to data from the KiGGS Study, which serves as a reference for German children [30]. The KiGGS Study includes data from both healthy children and children living with obesity, allowing for a comprehensive comparison and assessment of HrQoL among different groups.

### 2.4. Data Analysis

Demographical and anthropometric data such as sex, weight, height, BMI, age, and laboratory data like total cholesterol, glutamatpyruttransaminasis (GPT), HbA1c, homeostatic model assessment for insulin resistance (HOMA index), were retrospectively assessed using patients’ clinical charts. A statistical data analysis was performed using SPSS 24 computer software (IBM Corporation. IBM Statistics for Windows. Amonyk, NY, USA). A significance level of 0.05 was applied throughout the presented data. Descriptive data analyses included calculation of frequencies, means, median, standard deviation, and 95% confidence interval (95% CI). Independent T-tests were employed to assess the differences in eating behavior, measured by the K-FEV questionnaire. The tests were performed, yielding t-statistics, degrees of freedom and *p*-values for eachsex category, as mentiond before. A Wilcoxon rank sum test was performed to compare the differences between the variables measured in those two cohorts. For analysis of variance, results for the groups in the pre-COVID-19 cohort (June 2017 to June 2019) were compared with results for the COVID-19 cohort (June 2020 to June 2022). To assess the comparability of the two cohorts, we employed the Kolmogorov–Smirnov (K-S) test, a nonparametric statistical test, using SPSS software. The K-S test was applied to examine the distributional differences in terms of cohort size, sex distribution, age distribution, migrational status, and proportion of non-Caucasians. A significance level of α = 0.05 was used to determine the statistical significance of the results. If the obtained *p*-value from the K-S test was greater than 0.05, this indicated no significant difference between the compared distributions.

## 3. Results

### 3.1. Cohort Characteristics

A total of 264 children and adolescents (mean age 13.0 ± 4.1 years; female 47.6%) with obesity (mean BMI z score 2.44 ± 0.69) were recruited during the COVID-19 observation period (from June 2020 to June 2022). In the pre-COVID-19 comparison period, 265 children/adolescents (mean age 12.9 ± 4.1 years; female 52.0%) were examined. The visits in both cohorts were recruitments for the obesity treatment program. A total of 54.42% of patients in the COVID-19 cohort had a migrant background, and 57.20% of participants in the pre-COVID-19 cohort had a migrant background. The K-S test revealed no statistically significant differences (*p* > 0.05) in cohort size, sex distribution, age distribution, migrational status, or proportion of non-Caucasians. In conclusion, our analysis revealed that there were no significant differences in the aforementioned parameters between the two cohorts, suggesting that the two cohorts can be considered comparable in these aspects, and ensuring a robust basis for further analyses and interpretations.

Daily media time was reported to be significantly higher (*p* < 0.01) during the COVID-19 pandemic. Laboratory analysis revealed a significant increase in the HOMA index (*p* < 0.01), total cholesterol (*p* < 0.01), and GPT (*p* < 0.01) in the COVID-19 cohort compared with Pre-COVID-19. All individuals had normal HbA1C levels in both cohorts (5.40 vs. 5.31). Data are presented in Table 1. 

### 3.2. Eating Habits

#### 3.2.1. Food Content and Food Frequency

The study found that participants in the COVID-19 cohort had a significantly higher intake and an unhealthy consumption of obesity-promoting food components or meal portions compared to those in the pre-pandemic period. The Wilcoxon rank sum test demonstrated the significance of these findings at a *p*-value of less than 0.005. Specifically, the COVID-19 cohort reported a significant increase in the consumption of soft drinks, meat and sausages, fast food and delivery foods, chocolate and sweets, chips, salted nuts and snacks, gummy bears, and wine gums. However, in the area of cakes, pies, and cookies, a significantly decreased frequency of consumption was reported in the COVID-19 compared to the pre-COVID-19 group. Furthermore, patients in the COVID-19 group reported a reduced consumption of foods beneficial for weight development, including significantly less mineral water, tea and water, fresh fruits, and vegetables, when compared to the pre-COVID-19 period. Detailed data and interpretations can be found in Table 2, which summarizes the findings related to the consumption patterns of obesity-promoting food components and meal portions in the two cohorts. 

#### 3.2.2. Nutritional Behavior

When compared to those in the pre-pandemic period, both male and female children and adolescents in the COVID-19 group had significant differences in all three scale domains of the studied eating behavior. The mean cognitive hunger-control scores were as follows: in the pre-COVID-19 cohort, male 11.08, female 9.88, total 10.11; in the COVID-19 cohort, male 8.71, female 8.88, total 8.78. These results were found to be statistically significant (*p* < 0.05) compared to both the pre-COVID-19 cohort and the reference data in male adolescents. The reference data showed a higher mean score, with male 13.07, female 10.62, total 11.84, and significantly differed from the COVID-19 cohort (females and total). The lower scores for the COVID-19 pandemic indicate more spontaneous and unrestrained eating behavior among adolescents with obesity during the pandemic. For the variable “eating-associated disruptibility”, significant differences were revealed between the cohorts. In the pre-COVID-19 cohort, the mean values were 9.11 for males, 9.01 for females, and 9.05 for the total group. However, in the COVID-19 cohort, the mean values increased to 12.31 for males, 13.24 for females, and 12.78 for the total cohort (*p* <0.005). These results suggest a higher disturabability while eating in the COVID-19 cohort compared to the pre-COVID-19 cohort and the reference data, where the mean values were 7.12 for males, 8.47 for females, and 7.80 for the total group. The results for the variable “hunger sensations while fasting” indicate that there were no significant differences between the COVID-19 cohort and the pre-COVID-19 cohort. In the pre-COVID-19 cohort, the mean values were 12.10 for males, 9.01 for females, and 10.40 for the total group. Similarly, in the COVID-19 cohort, the mean values were 10.10 for males, 9.11 for females, and 9.75 for the total group. However, when comparing both cohorts to the reference data, there were significant differences (*p* < 0.05). The reference data showed lower mean values of 5.72 for males, 6.28 for females, and 6.00 for the total group. These findings suggest that high values in this section are associated with a higher degree of discomfort during fasting situations.

**Figure 1:**
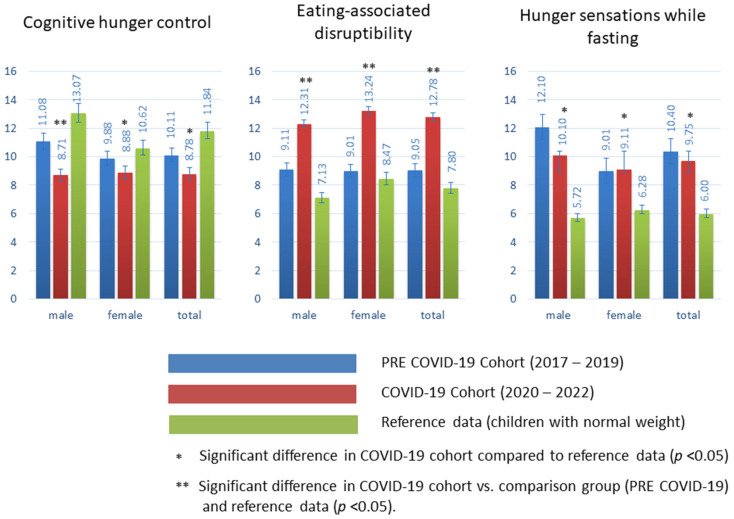
Sub-areas of obesity-promoting nutritional behavior; comparison of the COVID-19 cohort (medium gray), the pre-COVID group (dark gray) and normal-weight children (light grey) [22].

These findings suggest a trend towards obesity-promoting nutritional behavior in all sub-areas of the COVID-19 cohort compared to the pre-COVID.19 group. The findings are demonstrated in Figure 1. 

#### 3.2.3. Health-Related Quality of Life (HR QoL)

**Figure 2:**
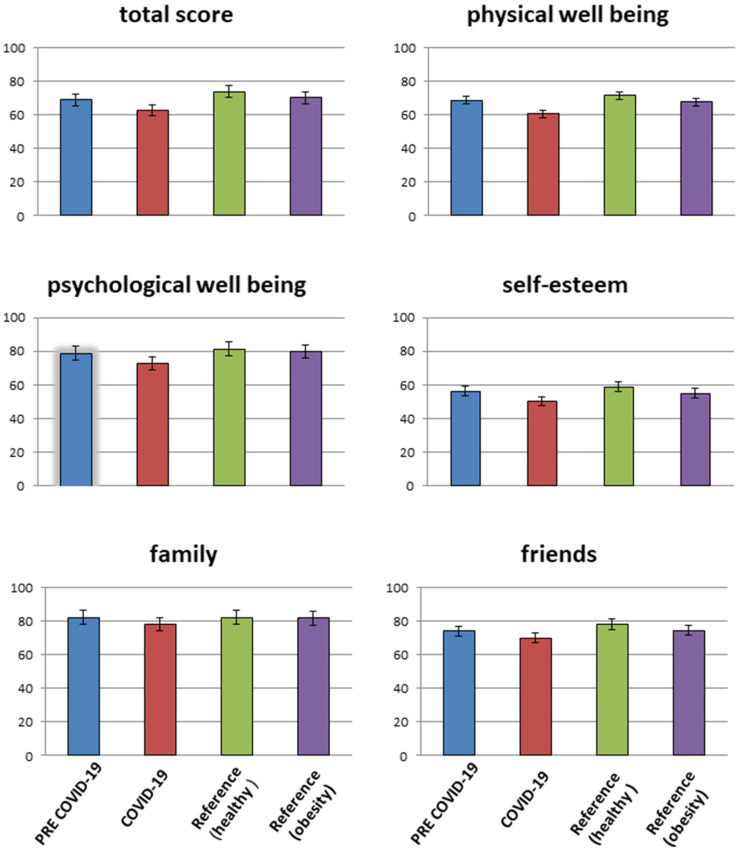
Total score (except the school subscale) and subdomains of HRQoL compared between the COVID-19 cohort (light gray), the pre-COVID group (medium light gray), reference data from normal-weight children (medium–dark grey) and reference data from overweight children (dark grey) [26,27].

Compared with referral data from normal-weight children and adolescents, the observation group and comparison cohort showed a decreased quality of life in all assessed domains (Figure 2). The data of the pre-pandemic comparison group were similar to the reference values of the KIGGS study of children/adolescents with obesity (20). Adolescents who were part of the COVID-19 cohort had significantly (*p* = 0.001) lowered parameters for “physical well-being”, “psychological well-being”, “self-esteem” and “friends” in the KINDL-R questionnaire when compared to the pre-pandemic period and the reference data from the KIGGS study. Only the decrease in the domain of “family” in the HrQoL did not reach statistical significance. A sub-analysis showed comparable parameters over the entire COVID-19 period (Table 1).

## 4. Discussion

This study shows that the obesity-promoting dietary behaviors and food choices of children referred to an obesity tretament program were further increased during the COVID-19 pandemic. In addition, the health-related quality of life of children and adolescents with preexisting obesity was also lower compared to the time before the pandemic. While there were no significant changes in anthropometric data, these changes in dietary behavior may lead to further weight gain and the progression of obesity-associated complications. 

The study also highlights the vulnerability of children and adolescents with pre-existing severe illnesses to the deterioration of their health conditions, especially in terms of mental health. These findings are consistent with a recent German population-based survey study [31,32,33,34,35]. Di Renzo et al., reported a population-based survey of daily lifestyle and dietary behaviors in Italy as early as April 2020, at the height of the first COVID-19 lockdown. Of the 3533 respondents (age 12–86 years; 76.1% female), 48.6% reported significant weight gain after this a short lockdown period, and changes in eating patterns toward an increased intake of sweets, pizza, cereals (some homemade), and a decrease in the consumption of fresh fruit, as well as an increase in the consumption of alcohol and delivery food [36]. A study by Pujia et al., also in Italy, investigated the effect of the entire first lockdown in the first half of 2020 on the dietary behavior of children and adolescents (439 participants) aged from 5 to 14 years by means of analog questionnaires. This study’s comes align with these findings, revealing an increase in the consumption of sweets, processed meat, bread, pizza and bakery products, as well as vegetables and fresh fruit. Children/adolescents with significant weight gain also showed a significantly increased consumption of food, especially nutrients, which encourage obesity [37]. 

It is possible that the pandemic-associated restrictions on social gatherings and events have impacted the consumption of baked goods such as cookies, cakes, and tarts, as described in this study. The disruption of daily routines, coupled with the absence of vital food sources like school meals and cafeterias, could be attributed to this shift. Despite the lengthy duration of the SARS-CoV-2 pandemic and the precautionary measures that were implemented, Germany, as well as most European nations and the U.S., was not able to provide a standardized food intake structure for young people. Consequently, many had to rely on fast food, delivery meals, and snacks. 

This alteration appears to be caused by the shift in previous structures in healthcare, everyday life, and education, which led to a decline in health-related quality of life and a significant transformation in dietary patterns. The HrQoL of children with obesity in the pre-COVID-19 comparison cohort was reported to be significantly lower than that in healthy children or children with other chronic diseases, such as asthma bronchiale. This is in alignment with other reports from the pre-pandemic era [29]. However, the SARS-CoV-2 pandemic had another impact on this phenomenon, leading to a further decrease in HrQoL in youth with obesity. This is consistent with results from other recently published studies on the pandemic [34,38,39,40]. The impact of the pandemic on health-related quality of life is more severe for adolescents with obesity compared to pre-existing healthy adolescents. This highlights their vulnerability to changes in daily life during the pandemic, which negatively affects their psychological, functional, physical, and social well-being. The pandemic has led to an increase in mental health problems, such as loneliness, anxiety, and uncertainty, which further aggravates the situation of these vulnerable children [41]. In Alabama, USA, a region with high pediatric obesity, a significant increase in new onset juvenile type 2 diabetes has been recently reported during the pandemic period [35]. 

Limitations: The present study is a retrospective, cross-sectional analysis conducted at a single center. As with any self-reported data, there is a risk of validity issues with regards to the eating behavior of the participants. However, the study included a high number of cases, which allows for an estimation of causal relationships. It is important to note that the study only included children with obesity who were referred to the obesity center by their pediatrician and had the self-motivation to attend consultations during the COVID-19 pandemic. This may introduce a selection bias since many chronically ill patients did not attend specialist outpatient clinics regularly during this time. While the study did not conduct an analysis of how socio-demographic factors affect dietary behavior and quality of life, the relatively good representation of various demographics in the study population, including gender and ethnicity, suggests that the findings may be applicable to the wider population of children with obesity in Germany [42]. 

## 5. Conclusions

This study provides evidence for differences in eating behavior among adolescents with obesity before and during the COVID-19 pandemic. In children refered to an obesity treatment program, the measures taken during the pandemic had negative consequences on their dietary habits and health-related quality of life (HrQoL). This first-of-its-kind investigation of this topic provides insights into the potential negative effects of the measures taken during the pandemic on this vulnerable population in an observational study. Disruptive social measures were associated with a unhealthier eating habits and a decrease in HrQoL in adolescents with obesity across genders and age groups.

In light of these findings, it is essential for policymakers, scientists, physicians, and other stakeholders to take proactive measures to address the potential impact of future societal changes, such as viral pandemics, on the dietary habits and quality of life of children and adolescents with obesity. It is important for our society to focus on programs for the secondary and tertiary prevention of the pandemic’s consequences on dietary behavior, food consumption, and HrQoL, especially for those who are already suffering from obesity.

## Figures and Tables

**Table 1:** Demographic parameters of the “Pre-COVID-19” and “COVID-19” cohorts.

	PRE-COVID-19 (June 2017–June 2019)			COVID-19 (June 2020–June 2022)			*p* * Values
Sex	♀	♂	total	♀	♂	total	
N (%)	138 (52.0)	127 (48.2)	265	125 (47.6)	139 (52.6)	264	ns
Age (years) [mean ± SD]	12.9 ± 4.1	13.4 ± 3.7	13.2 ± 4.0	13.3 ± 4.7	12.7 ± 4.2	13.0 ± 4.1	ns
Height (cm) [mean ± SD]	159.9 ± 11.4	168.1 ± 16.5		162.1 ± 12.0	161.5 ± 17.9		ns
Height z score	0.10	0.53		0.15	0.62		ns
Weight (kg) [mean ± SD]	72.7 ± 11.9	88.4 ± 27.1		79.7 ± 9.8	87.5 ± 11.2		ns
Weight z score	2.21 ± 0.27	2.34 ± 0.33	2.27 ± 0.35	2.33 ± 0.18	2.64 ± 0.19		ns
BMI (kg/m2) [mean ± SD]	29.8 ± 3.7	32.8 ± 4.1		30.03 ± 2.9	33.4 ± 4.3		ns
BMI z score	2.22 ± 0.39	2.28 ± 0.29		2.44 ± 0.69	2.65 ± 0.47		<0.05
Migration status (n; %)	87 (63,04)	79 (62.20)	166 (62.64)	68 (54.42)	83 (59.71	151 (57.20)	ns
Proportion non-caucasian (n; %)	78 (56.50%)	75 (37.95%)	153 (50.94%)	63 (50.4%)	81 (58.27%)	144 (54,54%)	ns
Daily screen time (minutes) [mean ± SD]	288 ± 41	302 ± 47	292 ± 43	337 ± 39	351 ± 54	348 ± 47	<0.01
HOMA index [95% CI]	3.1 [3.0; 3.7]	2.9 [2.7; 4.1]	3.2 [3.0; 4.6]	3.5 [3.2; 4.4]	4.2 [3.3; 5.1]	3.8 [3.3; 5.2]	<0.01
HbA1c (%; mmol/mol Hb)	5.27; 34.1	5,34; 34.86	5.31; 34.54	5.42; 35.74	5.39; 35.41	5.40; 35.52	ns
Cholesterol (mg/dL) [95% CI)	204.1 [187.4; 219.3]	194.2 [188.4; 222.2]	198.5 [168.0; 214.5]	207.2 [191.7; 229.5]	212.4 [189.9; 231.4]	208.8 [196.7; 219.5]	<0.01
GPT (U/l) [95% CI]	89.3 [56.7; 99.5]	63.3 [44.1; 79.9]	72.8 [51.2; 88.2]	102.4 [66.3; 109.5]	83.3 [67.7; 99.8]	93.4 [61.8; 101.2]	<0.01

*: *T*-tests/Wilcoxon test for independent samples; n: sample size; SD: standard deviation; CI: confidence interval; ns: not significant.

**Table 2:** Results of the food frequency survey (K- FFL) [22].

Food Content	Pre-COVID-19 (June 2017–June 2019) N = 265		COVID-19 (June 2020–June 2022) N = 264		Comparison between Pre-COVID-19 and COVID-19	
	Healthy Consumption Frequency n; % (95% CI)	Unhealthy Consumption Frequency n; % (95% CI)	Healthy Consumption Frequency n; % (95% CI)	Unhealthy Consumption Frequency n; % (95% CI)	*P* * (Healthy Consumption)	*P* * (Unhealthy Consumption)
Soft drinks	133; 50.3% (34.9–66.7%)	132; 49.7% (31.3–64.1%)	91; 34.5% (20.5–44.7%)	173; 65.5% (51.3–87.5%)	<0.05	<0.001
Mineral water, tea, water	183; 69.2% (51.8–80.2%)	82; 30.8% (19.2–49.8%)	133; 50.4% (37.0–67.8%)	131; 49.6% (28.2–61.2%)	<0.05	<0.001
Milk, cocoa	148; 55.9% (40.1–71.2%)	117; 44.1% (26.3–57.7%)	128; 48.4% (30.6–55.3%)	136; 51.6% (41.8–60.0%)	ns	*p* < 0.05
Yoghurt, buttermilk, curd	129; 48.5% (34.1–59.8%)	136; 51.5% (34.3–61.2%)	133; 50.3% (37.9–66.7%)	131; 49.7% (38.0–59.8%)	ns	ns
Cheese	120; 45.3% (31.4–57.8%)	145; 54.7% (37.9–72.5%)	158; 59.8% (48.5–72.2%)	106; 40.2% (31.2–57.8%)	<0.05	<0.05
Meat/suasages	74; 27.8% (11.2–45.3%)	191; 72.2% (56.4–87.8%)	34; 12.8% (2.5–31.2%)	230; 87.2% (62.9–95.4%)	<0.05	<0.01
Fish	23; 8.7% (1.9–16.5%)	242; 91.3% (84.8–99.9%)	10; 3.7% (0.4–8.2%)	254; 96.3% (82.8–99.9%)	<0.05	ns
Mixed bar, white bread, rolls	126; 47.4% (33.6–61.2%)	139; 52.6% (33.1–63.7%)	154; 58.4% (41.1–70.7%)	110; 41.6% (37.9–66.0%)	ns	ns
Wholemeal bread, wholemeal rolls	74; 27.8% (14.9–44.7%)	191; 72.2% (57.9–82.1%)	62; 23.5% (11.7–45.2%)	202; 76.5% (57.2–82.1%)	ns	ns
Rice/pasta	76; 28.7% (14.7–42.6%)	189; 72.3% (56.2–88.2%)	28; 10.7% (2.8–22.1%)	236; 89.3 (76.7–98.6%)	ns	<0.05
Fresh fruit	100; 37.9% (19.9–44.3%)	165; 62.1% (45.0–76.3%)	166; 62.9% (45.1–78.5%)	98; 37.1% (13.8–47.9%)	<0.05	<0.05
Vegetables/fresh/frozen)	97; 36.5% (23.1–51.4%)	168; 63.5% (44.2–71.9%)	28; 10.5% (2.8–20.1%)	236; 89.5% (77.6–98.4%)	<0.05	<0.01
Fast food/delivery food	83, 31.2% (24.6–43.2%)	182; 68.8% (58.3–77.8%)	12; 4.7% (0.1–10.9%)	252; 95.3% (87.8–99.9%)	<0.05	<0.01
Chocolate/sweets	61; 22.9% (11.6– 30.2%)	204; 77.1% (51.2–85.4%)	21; 7.8% (3.2–16.4%)	243; 92.2% (69.7–98.9%)	<0.05	<0.001
Chips, salty nuts, snack biscuits	77; 28.9% (20.7–39.8%)	188; 71.1% (59.9–78.5%)	11; 4.2% (0.0–14.2%)	253; 95.8% (87.3–99.9%)	<0.05	<0.01
Gummi bears, wine gum	100; 37.8% (24.3–55.1%)	165; 62.2% (47.2–73.0%)	36; 13.8% (5.4–17.1%)	228; 86.2% (78.0% -92.1%)	<0.05	<0.01
Cakes, tarts, biscuits	145; 54.7% (39.8–70.1%)	120; 45.3% (42.6–67.1%)	208; 78.9% (66.1–88.4%)	56; 21.1% (8.7–34.8%)	<0.05	<0.001

*: Wilcoxon rank sum test for independent samples; n: sample size; SD: standard deviation; CI: confidence interval; ns: not significant.

## Data Availability

The data presented are part of the KiCK program. Data collection and analysis were performed at the Center for Clinical Studies of the Children’s Hospital AUF DER BULT, Hanover, with support from the Department of Medical Psychology at Hanover Medical School. All data presented are available as source data and can be provided if desired.

## Supplementary Materials

The following supporting information can be downloaded at: https://www.mdpi.com/article/10.3390/nu15132936/s1, Table S1: Contents of the scales of the K-FEV questionnaire [16,17].

## Abbreviations

SARS-CoV-2	Severe-acute-respiratory-syndrome-related Coronavirus 2
COVID-19	Coronavirus disease 2019
BMI	Body mass index
SDS	Standard deviation score
HrQoL	Health-related quality of life
HOMA	Homeostasis Model Assessment
