# Supplementary material for: Alterations in Dietary Behavior, Appetite Regulation, and Health-Related Quality of Life in Youth with Obesity in Germany during the COVID-19 Pandemic
Source: Nutrients. 2023 Jun 28;15(13):2936. doi: 10.3390/nu15132936 (PMC10346875; doi:10.3390/nu15132936)
Supplement: Supplementary file 1 [file nutrients-15-02936-s001.zip › nutrients-2445824-supplementary.pdf]

Supplementary Table S1: Contents of the scales of the K -FEV questionnaire [16,17].

Scale	Content
**Cognitive hunger control** (21 items)	This section captures cognitive hunger control in terms of restrained eating behavior is aimed at restricting food intake (mostly from a calorie perspective). The goal of restricted food intake is always weight control (weight loss or avoidance of weight gain)
**Disruptibility of eating behavior** (16 items)	Assessment of the extent to which eating behavior can be disturbed by situational stimuli (external stimuli such as smell, sight of food) or the patient's emotional state (anxiety, distress). In the case of restrained eating behavior, this dimension characterizes a disinhibition of the exercised cognitive control by situational factors. In the case of unrestrained eating behavior without corresponding cognitive control, aspects such as satisfaction of eating needs and eating motivation are inquired about
**Hunger sensations while fasting** (14 items)	Recording of subjectively experienced feelings of hunger and their behavioral correlates during periods without food intake
